# MPD: multiplex primer design for next-generation targeted sequencing

**DOI:** 10.1186/s12859-016-1453-3

**Published:** 2017-01-05

**Authors:** Thomas S. Wingo, Alex Kotlar, David J. Cutler

**Affiliations:** 1 Division of Neurology, Atlanta VA Medical Center, Decatur, 30033 GA USA; 2 Department of Neurology, Emory University School of Medicine, Atlanta, 30322 GA USA; 3 Department of Human Genetics, Emory University School of Medicine, 615 Michael Street NE, Atlanta, GA 30322 USA

**Keywords:** DNA-sequencing, Next-generation sequencing, Primer design, Targeted resequencing

## Abstract

**Background:**

Targeted resequencing offers a cost-effective alternative to whole-genome and whole-exome sequencing when investigating regions known to be associated with a trait or disease. There are a number of approaches to targeted resequencing, including microfluidic PCR amplification, which may be enhanced by multiplex PCR. Currently, there is no open-source software that can design next-generation multiplex PCR experiments that ensures primers are unique at a genome-level and efficiently pools compatible primers.

**Results:**

We present MPD, a software package that automates the design of multiplex PCR primers for next-generation sequencing. The core of MPD is implemented in C for speed and uses a hashed genome to ensure primer uniqueness, avoids placing primers over sites of known variation, and efficiently pools compatible primers. A JavaScript web application (http://multiplexprimer.io) utilizing the MPD Perl package provides a convenient platform for users to make designs. Using a realistic set of genes identified by genome-wide association studies (GWAS), we achieve 90% coverage of all exonic regions using stringent design criteria. Using the first 47 primer pools for wet-lab validation, we sequenced ~25Kb at 99.7% completeness with a mean coverage of 300X among 313 samples simultaneously and identified 224 variants. The number and nature of variants we observe are consistent with high quality sequencing.

**Conclusions:**

MPD can successfully design multiplex PCR experiments suitable for next-generation sequencing, and simplifies retooling targeted resequencing pipelines to focus on new targets as new genetic evidence emerges.

## Background

The advent of next-generation sequencing has allowed for an unprecedented study of how genomic variants, particularly those in coding regions influence traits and disease. Currently, whole-exome and whole-genome sequencing remain prohibitively costly for studying a few genetic loci in hundreds to thousands of individuals, which might be the design of a typical validation experiment for genome-wide association studies (GWAS). A number of resequencing strategies exist for such validation experiments and several rely on multiplex PCR to capture many loci in a single PCR reaction.

Multiplex PCR is a technique that allows for simultaneous amplification of two or more loci using PCR primer pairs that are predicted to not interfere with each other within the reaction [1]. Traditionally, multiplex PCR products were isolated by size, purified and sequenced independently; however, coupling multiplex PCR with barcoding of samples and next-generation sequencing is a powerful technique to rapidly isolate and sequence multiple regions simultaneously in many samples using the 48.48 Access Array System (Fluidigum Corp., San Francisco, CA, USA). The main hurtle of coupling multiplex PCR with next-generation sequencing is efficient primer design. A robust solution is one that identifies primers that anneal to a single place in the genome and combines those primers into compatible groups. Compatible primers are those with similar GC content, T_m_, amplicon size, and amplicons that do not target overlapping regions. Existing tools are not well suited for this purpose because they focus on either a small portion of DNA for the primer design, require post-processing of primers to create pools or are tailored for epigenetic analysis, or require post-processing to create compatible pools [2–4]. Here, we present MPD, a software package designed with the aforementioned requirements in mind, that automates the design of multiplex PCR primers for next-generation sequencing of genomic DNA (Table 1).

**Table 1:** Comparison of Multiplex Primer Design software to existing primer design software

	Software					
Feature	MPD	BatchPrimer3	MPprimer	PCRTiler	Primer-BLAST	PrimerPlex
Multiple overlapping amplicons for genomic regions	Yes	No	No	Yes	No	No
SNP-specific amplicons	Yes	No	No	No	No	Yes
Multiplex compatible primers	Yes	No	No	No	No	Yes
Input	BED	Fasta sequence	Fasta sequence	Fasta sequence	Fasta sequence	dbSNP id or Fasta sequence
License	GNU GPL v3	GNU GPL v2	GNU GPL v3	GNU GPL v3	GNU GPL v2	Comercial

## Implementation

Multiplex PCR Design (MPD) software consists of a C library and programs used to design and pool compatible primers and a Perl package that provides convenience functions for sanitizing inputs, executing and processing the C programs, and summarizing results. To minimize human error, the package can write specifically formatted files to enable bulk oligonucleotide ordering via direct upload and addition of appropriate adapters to primers for compatibility with the 48.48 Access Array System.

The MPD C program designs primers using k-mers in a similar fashion to how BLAT finds compatible sequences [5]. It takes a specially prepared hashed version of the genome, flat dbSNP files, standard PCR parameters, and a bed file of target regions. All possible primers that cover a user-specified region are examined. Primers are immediately excluded if any of the following is true: 1) they form hairpins, 2) dimerize to each other, 3) have T_m_ outside the user specified range, 4) have GC content outside the user specified range, 5) occur within a repeat-masked region of the genome, 6) overlap a high frequency SNP, or 7) if the last 7 bases of the primer anneal within the amplified product. T_m_ and other primer characteristics were calculated using established algorithms [6]. Primers not rejected for any of these criteria are given a “quality score” which is an estimate of the primers commonness within the genome. Smaller scores represent primers with less common subsequences within them. A score of 1 would indicate that every k-mer of size 15 or smaller within the primer was absolutely unique, which is not actually possible, but scores near 1 indicate that most k-mers of size 14–15 are nearly unique. Primers with non-unique 15mers at the 3’ end of the primer are given large penalties. After all primers have been identified compatible with the supplied specification, a matrix of compatibility is created, and primer pairs are determined to be compatible if all of the following are true: 1) no primer dimerizes with another, 2) all primers have T_m_’s within 2 °C, 3) primer pairs do not target overlapping regions, and 4) amplified regions are within 20% of the maximum allowable amplicons size of one another (usually, 20–30 bp). The final criterion is important to avoid race conditions where smaller amplicons predominate the reaction. Pooling begins by either selecting the primer compatible with the most or least primers and proceeds recursively until all compatible primers are pooled.

**Fig. 1:**
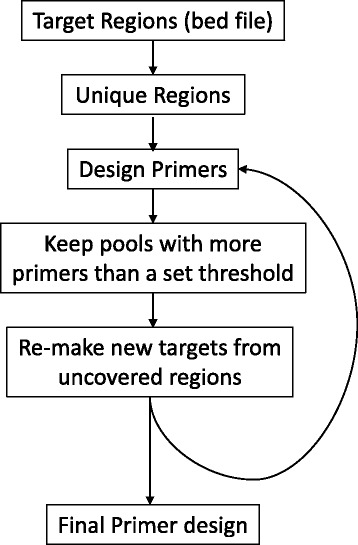
Example pipeline for primer design. Each successive iteration of the loop would loosen PCR design parameters by increasing the acceptable T_m_ or amplicons size up to a set limit or maximum iterations

The MPD Perl package offers convenience functions to process bed files into unique regions, launch and process the MPD C program output, and check MPD primers against a local compiled version of isPcr (UCSC genome browser’s *in silico* PCR tool [7]). Figure 1 and an included example script demonstrates the most common usage: a configuration file and target bed file are supplied, the bed file is sanitized to the unique regions, and primer pools are created that match the design specifications on the first iteration. Primers above a set threshold are retained, and optional additional iterations are made to loosen PCR parameters up to a set threshold. Optionally, isPcr may be used to provide an orthogonal validation for PCR primer uniqueness and genomic coordinates. After the final PCR pool design, all primers are written to (1) a plain text file, (2) a file suitable for use with isPcr, and (3) an excel file that is suitable for upload for batch synthesis of oligonucleotides in 96-well plate format. Additionally, a coverage file is provided indicating which primer(s) cover what target regions. To facilitate use with the 48.48 Access Array System, required forward and reverse primer sequencing adapters may be optionally added.

### Web application

The full Multiplex Primer Design (MPD) program is accessible online (http://multiplexprimer.io). The web application allows users to submit primer design jobs by uploading a list of coordinates to amplify as a simple bed file. Once the bed file is uploaded, the web server submits the data to a job queue, waiting until a worker hosted on Amazon’s EC2 cloud computer platform is available to run the job. Under typical conditions this happens within seconds. Once a worker reserves a job, it sends real-time progress updates back to the browser, allowing the user to monitor the progress of the primer design submission from anywhere in the world. Users may also opt-in to email notifications of major state changes, such as primer design success. Once completed, output files may be downloaded, and the design summary may be viewed directly in the browser.

### Genomic DNA samples

Human DNA samples used in this study were provided by the Emory Alzheimer’s Disease Research Center (ADRC), which recruits community volunteers for studies of aging and memory. Genomic DNA was extracted from human blood using the Gentra Puregene Blood kit (Qiagen) following the manufacturer’s protocol.

### Primer design and capture

MPD was used to design primers for all exonic regions of the following genes: *ABCA7*, *APOE*, *BIN1*, *CD2AP*, *CD33*, *CLU*, *MS4A6A*, and *PICALM* using conditions recommended for the Access Array System and compatible for sequencing on an Illumina MiSeq. For validation purposes, we restricted our analysis to the first 47 pools identified so only 1 Access Array System would be required per 48 samples. The primers were synthesized on 6 plates using standard desalting and normalized to 60 mM concentration with the appropriate forward and reverse adapters added to the respective primers. Individual primers were pooled and amplification of 48 samples of genomic DNA was performed using the Access Array as per manufacturer’s protocol. All samples were barcoded according to the manufacturer’s protocol and 250 bp paired-ended sequencing was performed on an Illumina MiSeq. Of note, the forward and reverse sequencing adapters add about 100 bp of sequence to the resultant amplicons.

### Primer design validation

All raw fastq files were mapped against hg38 build of the human genome using PE Mapper (https://github.com/wingolab-org/pecaller) and trimmed by 27 bp, which is 1 bp larger than the longest primer, from the 5’ end of the read to avoid sequencing the primers directly. Base calling and variant detection was performed using PE Caller with default parameters (theta = 0.001, probability to call = 0.95), and annotation was performed using SeqAnt [8].

Quality control was performed in 2 phases. First, samples were examined within groups that underwent capture together. Primer regions with >3 SD missing sites were dropped from all samples, and samples with >3 SD missing data were likewise excluded. Second, samples from all batches were combined, and those with >3 SD missing data or excess heterozygosity were dropped. Reported sites are those with >95% completeness, and variant sites that failed Hardy-Weinberg filtering at 10^-7^ were excluded; however, no site failed Hardy-Weinberg filtering.

## Results

For the 8 genes, we designed 330 primer pairs in 107 primer pools with an average of 2.4 primers per pool (range: 1–7). The primer pairs cover 90% (24,916 bp/27,657 bp) of the targeted bases and cover a total of 43,646 bp total because regions flanking the target are covered. The mean *in silico* amplicon size was 277 bp (247–300 bp) with a mean primer T_m_ of 60.2C (54.75–62.96C) and length of 20.8 bp (17–26 bp). We selected 47 primer pools (175 primer pairs) for wet-lab testing because they could be amplified on a single Access Array chip.

Of the 326 samples, 13 samples were excluded due to low coverage and a median of 22 primer pairs were dropped per batch due to low coverage (range 16–24). Of the failed primers, 13 failed in a single batch, which does not preclude usable data across the entire experiment whereas 18 failed in over half the batches which does. We note that the T_m_ and GC content was higher among failed primer pairs with a mean T_m_ of 62.11 °C whereas working primers had a mean of T_m_ 60.36 °C, which was statistically significant (T = 3.7063, *p* = 0.0005). The GC content of the failed reactions also tended to be higher 0.55 versus 0.50 although this was not significantly different (T = 1.7207, *p* = 0.096). The spearman rank correlation between the number of primers in a pool and the number of failed primers was -0.2894 (*p* = 0.04847) indicating that failed primers tend to occur in smaller pools suggesting that increased pool size does not lead to higher primer failure.

An average of 25,205 bp were sequenced per individual at 99.7% completeness with a mean depth of coverage of 300X (104-441X). A total of 16,295 bp were sequenced in the original targeted regions. Among the region of interest, we identified 207 SNPs, 6 insertions and 11 deletions total with each sample averaging 11.8 SNPs with 4.6 replacement and 3.6 silent sites. The overall average transition to transversion ratio was 3.16 per individual and silent to replacement ratio was 0.89. The mean minor allele frequency of 0.023 ± 0.072 for variant sites (range 0.0016–0.4313). We found dbSNP entries for 62 and 78% of the replacement and silent sites, respectively.

## Discussion

We demonstrate the MPD software is well suited to designing targeted resequencing experiments for use with the Access Array System (Fluidigm, San Francisco, CA, USA). We resequenced a realistic collection of genes that were proposed as candidate regions by Alzheimer’s Disease GWAS [9]. For these genes MPD was able to design primers over the majority of regions targeted, even particularly challenging regions with repetitive regions and high GC content (e.g., *APOE*). Importantly, the design primer pairs performed well in wet-lab testing. It is hard to directly compare the number of variants observed in our experiment to those observed through whole-exome or whole-genome sequencing because of the relatively few expected number of variants we ought to observe per subject. Most large-scale sequencing projects are interrogating millions of sites so their estimates of transition to transversion or silent to replacement ratios are more reliable than an experiment that targets only 25Kb. Despite this, the number and nature of variants we observe are consistent with high quality sequencing and compares favorably to whole-exome experiments when considering small regions in isolation. The weaknesses of multiplex PCR for next-generation sequencing are similar to those inherent in PCR-based capture methods, namely, the region must contain unique genomic sequence and not be enriched for high GC content.

Our approach for primer pooling differs from recommendations by Fluidigm in two ways. The first recommendation is that primers within a pool should be within 20% of the average amplicon size of the pool. Our software allows primers to be compatible if their amplicons are within 20% of the maximum amplicon length (e.g., for a 400 bp amplicon this threshold would be set at 80 bp) which achieves nearly the same goal that one set of amplicons dominate the reaction. The second recommendation is that pairs within a pool must anneal to targets separated by at least 5 kb. Our software checks for any annealing of amplicons within a pool to avoid interaction of amplicons within a pool, which we suppose is the intention behind the recommendation.

## Conclusion

The MPD software is able to design multiplex PCR experiments suitable for next-generation targeted resequencing. The software allows an iterative design approach where initially stringent conditions and subsequently loosened to maximize the number of high-quality primers that are as close to the initial design criteria as biologically feasible. The MPD software coupled with the 48.48 Access Array System are well-positioned for sequencing 10-100Kb per sample on hundreds to thousands of samples and may be quickly retooled to enable shift in targeted genes as new genetic evidence emerges.

## Availability and requirements

- Project name: MPD
- Project home page: https://wingolab-org.github.io/mpd-c/
- Operating system(s): Unix, Linux, OS X
- Programming language: C, Perl
- License: GPL (> = 3)
- Any restrictions to use by non-academics: None

## Abbreviations

GWAS: Genome-wide association study
MPD: Multiplex primer design
PCR: Polymerase chain reaction
